# Ten Simple Rules to Protect Your Intellectual Property

**DOI:** 10.1371/journal.pcbi.1002766

**Published:** 2012-11-08

**Authors:** Mark Jolly, Anthony C. Fletcher, Philip E. Bourne

**Affiliations:** 1 Brookes Batchelor LLP, London, United Kingdom; 2 Salix Management Consultants Ltd, London, United Kingdom; 3 Department of Pharmacology, University of California San Diego, La Jolla, California, United States of America; 4 Skaggs School of Pharmacy and Pharmaceutical Sciences, University of California San Diego, La Jolla, California, United States of America

The concepts that underpin the protection of ideas and inventions are not new; such laws have been around for several hundred years and are discussed under the broad heading of intellectual property (IP). IP is easily misunderstood, but at the same time most scientists encounter it at some point in their career, as it is a necessary feature in the commercialization of research.

The term intellectual property includes such concepts and rights as copyright, trademarks, industrial design rights, and patents. It is important to remember that IP is a tool to help your endeavours, and not a goal in itself. Having IP for its own sake is pointless. IP can be crucial in commercializing research and running a successful science-based business, but having a patent and having a successful patented product are two very different things.

Above all, IP can only work for you if you understand what it is, why you want it, and what you are going to do with it. These ten simple rules are intended to provide an overview of these issues; however, we must start with a warning. Laws relating to IP change all the time, they are complex, sometimes rather obscure, and are very different from country to country. For example, research surrounding methods of treatment by surgery and therapy and diagnostic methods are patentable in the United States, but specifically excluded from patentability in Europe [1]. However, these boundaries seem to be shifting in both the US and Europe. In short, we are dealing with a complex and changing subject and restrict ourselves here to the guiding principles.

## Rule 1: Get Professional Help

Although the process of obtaining IP looks deceptively simple, like many things the devil is in the detail. Let's consider patents as an example. The practicalities of patent application are straightforward; you simply file documents with the relevant body indicating that a patent is sought, and provide the identity of the person applying and a description of the “invention” for which a patent is sought. The patent office will then write back to you with an application number.

However, there is no guarantee that a patent application will become a granted patent. Indeed, at the application stage they do not even check that your description describes an invention at all. Even if you draft a description in as much detail as you would for an academic research paper and file it yourself, the prospect that it will be granted and enforceable is very low. There is skill and technique, even a language, that patent attorneys and patent agents have that allows them to describe and define inventions in the way a patent office requires. As an example, in everyday parlance, the terms “comprise” and “consist” could be considered to mean the same, whereas they have very distinct meanings in a patent application.

The dangers are possibly even greater with trademarks and registered designs (also known as “design patents”)—these are generally granted with very little examination and patent offices are often even less inclined to suggest using a patent/trademark attorney for such “simpler” rights; however, the lack of examination means the validity of such a right is uncertain and they become open to challenge.

The costs of redrafting a self-filed application are invariably higher than the costs for drafting an application from scratch, and if there has been any disclosure it will probably not be possible to re-draft. So, in summary, if you want your IP to be valuable, you should seek professional advice at an early stage.

## Rule 2: Know Your (Intellectual Property) Rights

IP rights come in various guises, and each is a defensive right to pursue legal action in the event that a third party infringes. In very basic terms:

- Patents protect inventions—broadly, things that are new and not obvious—and the way they work. Sometimes this is expressed as “everything under the sun made by man”; however, there are numerous local exceptions from patentability—we touched on the complexities of methods of treatment above—but there are similar issues in relation to genes, computer programs, and business methods, for example.
- Registered designs protect the appearance of products (not the function, which is protected by patents).
- Trademarks protect brands (e.g., trade names and logos).
- Copyright protects the expression of ideas—i.e., the words you choose to use to describe your idea—not an idea itself.

Most businesses do not need the trinity of patents, trademarks, and designs; in fact, trademarks are probably the only IP most companies have or need, however for a few companies the full house is required: for example, consider the Apple® iPad®: two registered trademarks, a registered design for its shape, and of course patents for the way it interacts with the user. Not to mention copyright covering the code and the packaging. A huge battle in the courts around the world is currently taking place over these rights that may well effect changes in the law. The *Wall Street Journal* calls the recent Apple/Samsung case “the patent trial of the century” [2].

## Rule 3: Think about Why You Want IP (i.e., What You Will Actually Do with It)

Any money spent on IP is capital that cannot be spent on production, marketing, etc., so think carefully about why you are investing in protecting your IP. There are many good reasons: to stop people from copying you; to add value to your company if you want to sell it; to sell or license to a third party; to hold it in your armoury if you suspect you are going to be sued and want to countersue (for example, Google has spent a substantial amount of money buying patents recently [3]); even to reduce your tax bill (in certain countries profits attributed to patents can be taxed at a lower rate [3], [4]).

However, in general, IP is a right to prevent other people from doing something; owning IP does not necessarily give you the right to do anything yourself.

One school of thought says that IP is only valuable if you are willing to enforce or defend it, and the cost of such an action can be prohibitive. Indeed, the business model of “patent trolls” is to purchase patents, sometimes from those who cannot afford to enforce them, not to use the invention, but just to enforce against infringing companies. On the other hand, the term “defensive IP” has been used to describe IP obtained, not to stop other people from competing, but to stop a competitor from patenting something that you may wish to use in the future. Thus a patent application may be filed, and published but allowed to lapse, with no intention of ever enforcing it, simply because the step of publication will mean that should a competitor apply to patent the same or a similar invention, the patent office will locate your application and it will anticipate the competitor's application.

Note also that while this article is titled “Ten Simple Rules to Protect *Your* IP”, it is important not to be too introspective and to consider other people's IP. For example, successful strategies can be built around taking exclusive licenses—licenses that exclude even the IP owner from using the IP. One tactic to improve your competitive position can be to take an exclusive license under a patent, then either expand your range to include the patented product, or continue only to sell your own product, but use the exclusive license to prevent manufacture of the other by anybody else.

## Rule 4: If You Don't Protect the IP, Your Innovation Is Less Likely to Happen

Maybe you are not an entrepreneur yourself, but have an idea that you would like to see it exploited—it could, after all, make the world a better place. You can publish it—then anyone who wishes can use it freely. But the big question here is, will they? Many inventors think that by publishing their ideas freely they are more likely to have them exploited; however, the converse is often true (for example, in health care, where lack of patent protection is often cited as a major reason for not following up an idea (T. Roberts, former president of the Chartered Institute of Patent Attorneys [UK]).

The reason is economic: most innovations require investment, and investors look for a return on their money. However, ideas that are released without any IP protection will often immediately attract competitors who can perhaps undercut the inventor (for example, with economies of scale). This decreases the likelihood of investment in the development of an invention (which is often more crucial than the invention itself) and increases the need for investment in marketing, etc. to obtain a competitive edge.

So what we have to consider here is that—even if you don't want to profit personally from the innovation—it may still pay to protect it so that it will see the light of day through other investors. Remember, IP can be licensed and what happens to the resulting income is up to the IP's owner. And this is a point where it gets complex for scientists and others who invent as part of their employment. We will cover this in more detail in Rule 10.

## Rule 5: What's in a Name?

You have a great idea but it's not patentable, or you have applied for patent protection but are worried that it may not cover everything, and of course the protection will expire after 20 years [5]. This is where trademarks come in to fill the gap in your protection. Unlike patents and designs, a trademark or brand can be protected with a registration at any time (unless someone else has got there before you)—you do not need to have kept your name a secret, and once registered the right will only expire if you stop using it or fail to renew it (generally every 10 years). So, you can protect your invention with a patent and sell it under your brand, which is also protected. Once the patent protection expires, customers are used to buying your product with reference to your brand, and will hopefully continue to do so even though competitors may start offering rival products. Just make sure your brand is something memorable and unique to you.

Viagra is just one example of a trademark so closely associated with the product (sildenafil) that a good proportion of the market should remain in the hands of the trademark owner well after the patent has expired (in this instance, if priced competitively). You do need to be careful here in selecting the name you are protecting: descriptive brands are easy to market but hard to protect because descriptive terms do not fulfil the requirement of “distinct character”. And you can be too successful: many people now use the trademark Hoover to mean a generic vacuum cleaner, Thermos for a vacuum flask to keep food hot, or Tannoy for a public address system. It can be very expensive in terms of lawyers fees to police such trademarks and keep protecting these names and prevent them becoming simply part of the language and hence devalued.

## Rule 6: Be Realistic about What You Can, and Cannot, Protect

IP rights are, generally speaking, national rights provided by individual governments to regulate activity in that particular country. In some cases there are bilateral and multilateral agreements (for example, most of the world has signed up to the Berne Agreement, which accords the same level of copyright protection to foreign nationals of other Berne states that is provided to nationals of the state concerned [6]).

However, for most rights, it is a national issue. In an ideal world, each incremental improvement would be patented in each national jurisdiction (there are approximately 200 countries in the world), along with the name you trade under, and every brand would be the subject of a trademark, as would any color associated with your company and any sound you use, your products and their packaging would be the subject of registered designs, and your patent attorneys would be very wealthy!

In the real world it is essential to be realistic. A patenting regime covering more than the US, Europe, and a handful of other countries is a rare sight outside the realms of very large companies (such as big pharma), and even many big companies restrict themselves to key markets.

## Rule 7: It's Big Business and Controversial

The world of IP is a big one. It's controversial, as it has a huge impact on international relations and trade. It's also controversial for political reasons, as many people feel that aggressive protection stifles the utility of products that have the potential to do good in the emerging world (again, for example, big pharma). The World Intellectual Property Organization (WIPO) is the United Nations agency dedicated to this area [7], and it's worth considering its overarching aims, which include reducing the knowledge gap between developed and developing countries, and ensuring that the IP system continues to effectively serve its fundamental purpose of encouraging creativity and innovation in all countries.

Of course, many question the value to society of IP, or at least the expansion of IP, in promoting creativity and innovation. The Public Library of Science describes itself as a driving force of the open-access movement, and accordingly, unlike many copyrighted works, this article may be copied without seeking permission, provided that the original authors and source are cited.

It can be hard, for example, to defend the extension of copyright from 50 years after an author's death to 70 years on the grounds that the extra 20 years of protection is in any way likely to encourage creativity. Whatever your thoughts on IP, it is worth bearing in mind that others may disagree.

As a scientist and innovator you may be driven by many ideals: to make the world a better place, perhaps, or to buy yourself a yacht—we are all different. But like it or not, if you want to commercialize your ideas you cannot avoid the issue of IP, and we go back to Rule 1 here—get professional advice. Even if your aim is totally philanthropic you may still need to invest to protect your innovation, perversely because this is what will give it the biggest chance of actually succeeding. Simply make sure you tell your patent attorney what your ultimate aims are.

## Rule 8: Keep Your Idea Secret until You Have Filed a Patent Application

Little upsets a patent attorney more than hearing “I have a great idea—it's selling really well” or “I've shown it to a few companies and they seem very interested”.

There is an old maxim that says a secret shared is not a secret anymore. While a secret shared under a non-disclosure agreement (NDA)—documents most people have heard about but probably never read—ought to stay secret, discussing an invention under the umbrella of confidentiality is no substitute for being able to freely discuss or publish an idea that is protected by a patent application.

Obviously, once your idea is published by a journal it is too late to file a patent application—your invention has been made available to the public. However, earlier in the publication cycle the situation is different. If you send a paper to a journal for submission, it will (excluding open review) be treated as a confidential disclosure to the publisher and the reviewers. Notwithstanding, the best advice is still to file a patent application before submitting a paper, either to avoid a potential “abusive disclosure” or hold up the publication of the paper.

In summary, novelty is key to patentability and your own disclosures count against you, so remember to file a patent application *before* telling anybody who is not bound by confidence.

## Rule 9: Trade Secrets

Regarding patents, the economic reasoning behind the system is an exchange between you and the public. The government allows you a monopoly, and your side of the bargain is to disclose fully your invention so that once your 20 years of protection is up, it can be freely exploited for the good of society. A patent can provide you with a 20-year government approved monopoly. However, some ideas cannot be patented and indeed, some innovators don't want to patent their ideas. All is not lost here, however, as we fall back on an older idea and one much beloved of thriller writers: the trade secret.

If you really can keep a secret, your monopoly on an idea or product may never end. But once the genie's out of the bottle, like a champagne cork, you won't get it back in and you are unlikely to extract sufficient damages from whoever breaches confidentiality. Thus, if you have an idea that cannot be reverse engineered, you do not have to enter into the patent bargain. Trade secrets are free—just prevent the secret being disclosed. But bear in mind that that this can be very difficult indeed, but not impossible. Famous successful examples include the recipe for Coca-Cola and the formulation of the alcoholic beverage Chartreuse, which is only known by two monks.

## Rule 10: Make Sure the IP Is Owned in a Way That Allows Development

Notice that we don't suggest “make sure *you* own the IP of your invention”. If you discover something whilst working as an employee (e.g., of a company or an academic establishment), there will certainly be something in your contract about this. Generally, the employer will have first call on the invention, but may have clauses that will return rights to the individual if it is not exploited within a certain time—in some countries this is enshrined in law [8].

Ownership of IP is a minefield, and can be particularly difficult in an academic setting where numerous complicating features are involved. Universities, as employers, are likely to have a right to their employees' inventions; funding bodies may make their own claim; inventorship is not like authorship—the people whose names are on an academic paper are unlikely all to be inventors; and in cross-border collaborations, national laws on ownership may well be in competition with each other. One complicating factor that is often encountered is joint ownership: if you can, avoid joint ownership; instead, set up a company to own the IP and license it to partners if necessary (otherwise you face differing national rules on what joint owners can do with and without each other's permission).

If it is necessary to share IP, work out at the beginning who owns what, what rights each party has and importantly who will have the right to future inventions. In fact this is a common theme in several of our Ten Simple Rules: as soon as money rears it ugly head, strife follows, so it's as well to plan for dispute resolution right from the beginning.

In summary, first, you can never act too early, but it's very easy to act too late. Like many topics that involve the law, IP is a mind-numbingly complex topic and more so, perhaps, as it's not national, but international, so get the very best professional advice you can. If you are working as an employee, speak to your company at the earliest stage; they have a vested interest in helping get it right. Second, because significant sums of money are involved, plan for future discord. Finally, persevere: your invention can make the world a better place.
